# Borderline gestational diabetes mellitus and pregnancy outcomes

**DOI:** 10.1186/1471-2393-8-31

**Published:** 2008-07-30

**Authors:** Hong Ju, Alice R Rumbold, Kristyn J Willson, Caroline A Crowther

**Affiliations:** 1 Discipline of Obstetrics and Gynaecology, The University of Adelaide, Women's and Children's Hospital, King William Road, North Adelaide, South Australia, 5006, Australia; 2 Menzies School of Health Research & Institute of Advanced Studies, Charles Darwin University, PO BOX 41096, Casuarina, Northern Territory, 0811, Australia; 3 Discipline of Public Health, The University of Adelaide, Adelaide, South Australia, 5005, Australia

## Abstract

**Background:**

The impact of borderline gestational diabetes mellitus (BGDM), defined as a positive oral glucose challenge test (OGCT) and normal oral glucose tolerance test (OGTT), on maternal and infant health is unclear. We assessed maternal and infant health outcomes in women with BGDM and compared these to women who had a normal OGCT screen for gestational diabetes.

**Methods:**

We compared demographic, obstetric and neonatal outcomes between women participating in the Australian Collaborative Trial of Supplements with antioxidants Vitamin C and Vitamin E to pregnant women for the prevention of pre-eclampsia (ACTS) who had BGDM and who screened negative on OGCT.

**Results:**

Women who had BGDM were older (mean difference 1.3 years, [95% confidence interval (CI) 0.3, 2.2], p = 0.01) and more likely to be obese (27.1% vs 14.1%, relative risk (RR) 1.92, [95% CI 1.41, 2.62], p < 0.0001) than women who screened negative on OGCT. The risk of adverse maternal outcome overall was higher (12.9% vs 8.1%, RR 1.59, [95% CI 1.00, 2.52], p = 0.05) in women with BGDM compared with women with a normal OGCT. Women with BGDM were more likely to develop pregnancy induced hypertension (17.9% vs 11.8%, RR 1.51, [95% CI 1.03, 2.20], p = 0.03), have a caesarean for fetal distress (17.1% vs 10.5%, RR 1.63, [95% CI 1.10, 2.41], p = 0.01), and require a longer postnatal hospital stay (mean difference 0.4 day, [95% CI 0.1, 0.7], p = 0.01) than those with a normal glucose tolerance.

Infants born to BGDM mothers were more likely to be born preterm (10.7% vs 6.4%, RR 1.68, [95% CI 1.00, 2.80], p = 0.05), have macrosomia (birthweight ≥4.5 kg) (4.3% vs 1.7%, RR 2.53, [95% CI 1.06, 6.03], p = 0.04), be admitted to the neonatal intensive care unit (NICU) (6.5% vs 3.0%, RR 2.18, [95% CI 1.09, 4.36], p = 0.03) or the neonatal nursery (40.3% vs 28.4%, RR 1.42, [95% CI 1.14, 1.76], p = 0.002), and have a longer hospital stay (p = 0.001). More infants in the BGDM group had Sarnat stage 2 or 3 neonatal encephalopathy (12.9% vs 7.8%, RR 1.65, [95% CI 1.04, 2.63], p = 0.03).

**Conclusion:**

Women with BGDM and their infants had an increased risk of adverse health outcomes compared with women with a negative OGCT. Intervention strategies to reduce the risks for these women and their infants need evaluation.

**Trial registration:**

Current Controlled Trials ISRCTN00416244

## Background

The prevalence of gestational diabetes mellitus (GDM) is increasing all over the world [1,2]. In Australia the recent prevalence estimates for GDM ranged from 5.2% to 8.8% [3]. The risks for both mothers with GDM and their infants are well-documented. For the infants, these include an increased risk of macrosomia, birth injuries such as shoulder dystocia, bone fracture and nerve palsies, hypoglycaemia, and hyperbilirubinaemia [4-7]. Women with GDM are at increased risk of developing pre-eclampsia and have an increased chance of need for induction of labour and caesarean section. Gestational diabetes is also a strong risk factor for later development of type 2 diabetes [8].

Although the risks associated with GDM are well recognised, the impact on maternal and infant health outcomes is less clear for borderline gestational diabetes mellitus (BGDM), which is characterised by values of glucose tolerance intermediate between normal and gestational diabetes. A recent 10 year audit examining the influence of different levels of glucose tolerance on pregnancy complications, [9] revealed a significantly increased risk of pre-eclampsia, caesarean section, neonatal hypoglycaemia and hyperbilirubinaemia for women with BGDM compared with women with normal glucose tolerance. The results are consistent with other literature reports, which identified an increasing risk of adverse maternal and infant outcomes with increasing plasma glucose values [10-12].

It is estimated that 6.6% of pregnant women or approximately 16,500 women have BGDM each year in Australia [9]. Given the uncertainty surrounding BGDM, we assessed data from participants in the Australian Collaborative Trial of Supplements with antioxidants Vitamin C and Vitamin E to pregnant women for the prevention of pre-eclampsia (ACTS) [13] to compare the maternal demographic, pregnancy and infant health outcomes of women who had BGDM (screened positive for GDM on oral glucose challenge test (OGCT) but their subsequent oral glucose tolerance test (OGTT) was normal) with women who screened negative on OGCT for GDM.

## Methods

The study population included women participating in the ACTS trial [13], a multi-centre randomised placebo controlled trial of antioxidant (vitamins C and E) supplements for the prevention of perinatal complications, who had an OGCT as screening for gestational diabetes. The methods and results of this trial have been reported previously [13]. Briefly, eligible women were: nulliparous, with a singleton pregnancy between 14 and 22 weeks of gestation with a normal blood pressure at the time of recruitment and who gave informed consent. Women with any of the following were ineligible: known multiple pregnancy, known lethal fetal anomaly, known thrombophilia, chronic renal failure, antihypertensive therapy or contraindication to vitamin C or E therapy including haemochromatosis or anticoagulant therapy. Randomisation was performed through a central telephone randomisation service. Women assigned to the vitamin group were provided a daily dose of 1000 mg vitamin C and 400 IU vitamin E until birth, and women in the control group were provided a matching placebo. An OGTT was offered between 24–30 weeks gestation, for those women who screened positive on OGCT test. The study protocol was approved by the research and ethics committees at the nine collaboration hospitals around Australia.

We compared demographic, obstetric and neonatal outcomes between women with BGDM and those who screened normal on OGCT. As the ACTS found no significant differences between the antioxidant and placebo groups for the risk of pre-eclampsia, intrauterine growth restriction or other serious outcomes for the infant, the analyses include the combined populations of women who received either antioxidant or placebo supplements.

### Data collection

Pregnancy outcome data including OGCT and OGTT results were collected prospectively from women's medical records. Sociodemographic variables were collected either from women's medical records or self-completed questionnaires at trial entry and included: maternal age, ethnicity, body mass index (BMI), social-economic status as measured by socio-economic index for area (SEIFA) score [14], maternal education, smoking status, blood pressure at trial entry, and family history of pre-eclampsia. Complete outcome data were available for all 1877 women randomised.

### Outcome variables

BGDM was defined as a positive OGCT (blood glucose ≥7.8 mmol/L 1 hour after a 50 g glucose load) and normal 75 g OGTT (fasting blood glucose <5.5 mmol/L and 2 hour blood glucose <7.8 mmol/L). Pregnancy outcomes assessed included: maternal adverse outcomes (a composite outcome defined as any of the following until six weeks postpartum: death, pulmonary oedema, eclampsia, stroke, thrombocytopenia, renal insufficiency, respiratory arrest, placental abruption, abnormal liver function, preterm prelabour rupture of membranes, major postpartum haemorrhage, postpartum pyrexia, pneumonia, deep-vein thrombosis, or pulmonary embolus requiring anticoagulant therapy) [13]; pregnancy induced hypertension (PIH); pre-eclampsia (defined as systolic blood pressure ≥140 mmHg or diastolic blood pressure [Korokoff V] ≥90 mmHg on at least two occasions four or more hours apart, or both arising after 20 weeks' gestation and one or more of the following: proteinuria, renal insufficiency, liver disease, neurological problems, haematologic disturbances, or fetal growth restriction) [15]; antenatal hospitalisation; preterm prelabour rupture of the membranes; induction of labour; mode of birth; postnatal complications such as postpartum haemorrhage and infection; and length of hospital stay.

Neonatal outcomes included a composite outcome of death or infant adverse outcome defined as: stillbirth or death of a liveborn infant before hospital discharge, birthweight <3^rd ^centile for gestational age, severe respiratory distress syndrome, chronic lung disease, intraventricular haemorrhage grade 3 or 4, cystic periventricular leukomalacia, retinopathy of prematurity grade 3 or 4, necrotizing enterocolitis, 5 minute Apgar score <4, seizures before 24 hours of age or requiring 2 or more drugs to control, hypotonia for ≥2 hours, stupor, decreased response to pain or coma, tube feeding for ≥4 days, care in the neonatal intensive care unit (NICU) >4 days, or use of ventilation for ≥24 hours [13]; gestational age at birth; preterm birth (<37 weeks); 5 minute Apgar score <7, infant body size at birth (weight, length and head circumference), small and large-for-gestational age (defined as a birth weight below the 10^th ^percentile or above 90^th ^percentile for gestation according to fetal sex on standardized birth-weight charts, respectively), macrosomia (defined as birthweight ≥4.5 kg), admission to NICU or neonatal nursery, respiratory distress syndrome, mechanical ventilation, antibiotics use after birth, encephalopathy (Sarnat 2 or 3 score) and length of hospital stay.

### Statistical analysis

Statistical analysis was carried out using SAS software, version 9.1. Dichotomous variables were analysed using log-binomial regression and presented as relative risks, with 95% confidence intervals; and continuous variables, if normally distributed, were analysed using Student's t-test and presented as mean differences, with 95% confidence intervals; non-parametric tests were used for skewed data. Analyses were then adjusted for maternal age and BMI given the strong association of these factors with GDM. A p value of 0.05 or less was considered to indicate statistical significance.

## Results

Of the 1877 women enrolled in the ACTS trial, 1804 (96%) did not have a fetal loss and underwent screening using a 50 g oral glucose challenge test for gestational diabetes. Of the women screened 1596 (88%) had a normal OGCT screening result, 68 (4%) had an abnormal OGTT and 140 (8%) had BGDM (screened positive on OGCT, normal OGTT).

Overall, women with BGDM and women with a normal OGCT had similar characteristics at entry to the study including ethnicity, socio-economic status and educational attainment (Table 1). Compared with women with a normal OGCT, women with BGDM were older (mean difference 1.3 years, [95%CI 0.3, 2.2], p = 0.01), less likely to have a normal BMI (RR 0.82, [95%CI 0.67, 0.99], p = 0.04) and almost twice as likely to be obese (RR 1.92, [95%CI 1.41, 2.62], p < 0.0001) (Table 1). There was no statistically significant difference found between these groups in the number of women who smoked or in their mean systolic or diastolic blood pressure at study entry.

**Table 1:** Demographics of women with borderline GDM compared with women with a normal OGCT

**Characteristics**	**Borderline GDM ** n = 140 (%)	**Normal OGCT ** n = 1596 (%)	**Relative risk ** [95% CI]	**p value**
**Age**^**a **^(years)	27.5 ± 5.4	26.3 ± 5.8	1.3 [0.3, 2.2]	0.01
**Race**				
Caucasian	129 (92.1)	1517 (95.1)	0.97 [0.92, 1.02]	0.22
Asian	5 (3.6)	47 (2.9)	1.21 [0.49, 3.00]	0.68
Other	6 (4.3)	32 (2.0)	2.14 [0.91, 5.02]	0.08
**BMI**				
Underweight (<18.5)	3 (2.3)	59 (4.0)	0.59 [0.19, 1.80]	0.34
Normal (18.5 – <25)	59 (45.7)	818 (55.9)	0.82 [0.67, 0.99]	0.04
Overweight (25 – <30)	32 (24.8)	380 (26.0)	0.96 [0.70, 1.31]	0.78
Obese (≥30)	35 (27.1)	207 (14.1)	1.92 [1.41, 2.62]	<0.0001
**SEIFA**^**b**^				
Low	40 (28.6)	429 (26.9)	1.06 [0.81, 1.40]	0.66
Low-Mid	25 (17.9)	288 (17.9)	1.00 [0.69, 1.44]	0.99
Mid-High	37 (26.4)	391 (24.5)	1.08 [0.81, 1.44]	0.61
High	38 (27.1)	490 (30.7)	0.88 [0.67, 1.17]	0.39
**Education**				
Secondary or lower	54 (39.7)	704 (45.0)	0.88 [0.71, 1.09]	0.25
TAFE or equivalent	38 (27.9)	361 (23.1)	1.21 [0.91, 1.61]	0.19
University	44 (32.4)	498 (31.9)	1.02 [0.79, 1.31]	0.91
**Smoking**	37 (26.4)	340 (21.3)	1.24 [0.93, 1.66]	0.15
**BP at trial entry**^**a **^(mmHg)				
Systolic BP	110.7 ± 11.2	110.1 ± 10.5	0.6 [-1.3, 2.4]	0.55
Diastolic BP	66.4 ± 9.0	65.3 ± 8.0	1.1 [-0.3, 2.5]	0.12

^a ^Values are mean ± standard deviation, and the comparisons are mean difference (95% CI)

^b ^Lower scores indicate lower socioeconomic status

BMI, Body mass index; BP, Blood pressure; SEIFA, Socio-economic index for area

In unadjusted analyses women with BGDM were more likely to experience a maternal adverse outcome (RR 1.59, [95%CI 1.00, 2.52], p = 0.05) and to develop pregnancy induced hypertension (RR 1.51, [95%CI 1.03, 2.20], p = 0.03) compared with women with a normal OGCT. These differences were not seen when adjustment was made for maternal age and BMI. There was no significant difference in the rate of pre-eclampsia between the two comparison groups (Table 2).

**Table 2:** Clinical outcomes among women with borderline GDM compared with women with a normal OGCT

**Outcome**	**Borderline GDM ** n = 140 (%)	**Normal OGCT ** n = 1596 (%)	**Unadjusted relative risk ** [95% CI]	**p value**	**Adjusted relative risk ** [95% CI]	**p value**
**Maternal adverse outcome**	18 (12.9)	129 (8.1)	1.59 [1.00, 2.52]	0.05	1.47 [0.92, 2.34]	0.11
**Pregnancy induced hypertension**	25 (17.9)	189 (11.8)	1.51 [1.03, 2.20]	0.03	1.31 [0.90, 1.90]	0.16
**Pre-eclampsia**	9 (6.4)	86 (5.4)	1.19 [0.61, 2.32]	0.60	1.08 [0.56. 2.10]	0.82
**Antenatal hospitalisation**	29 (20.7)	287 (18.0)	1.15 [0.82, 1.62]	0.42	1.17 [0.83, 1.65]	0.36
**PPROM**	6 (4.3)	41 (2.6)	1.67 [0.72, 3.86]	0.23	1.54 [0.66, 3.57]	0.32
**Induction of labour**	49 (35.0)	498 (31.2)	1.12 [0.88, 1.42]	0.34	1.06 [0.84. 1.34]	0.62
**Vaginal birth**	94 (67.1)	1184 (74.2)	0.90 [0.80, 1.02]	0.10	0.96 [0.86, 1.08]	0.48
Normal vaginal birth	70 (50.0)	865 (54.2)	0.92 [0.78, 1.10]	0.36	1.00 [0.85. 1.17]	1.00
Instrumental vaginal birth	24 (17.1)	319 (20.0)	0.86 [0.59, 1.25]	0.42	0.87 [0.60, 1.27]	0.48
**Caesarean section**	46 (32.9)	412 (25.8)	1.27 [0.99, 1.64]	0.06	1.13 [0.89. 1.43]	0.33
Elective	10 (7.1)	101 (6.3)	1.13 [0.60, 2.11]	0.70	1.01 [0.54, 1.88]	0.98
Emergency	36 (25.7)	311 (19.5)	1.32 [0.98, 1.78]	0.07	1.17 [0.87, 1.56]	0.30
**Caesarean section for fetal distress**	24 (17.1)	168 (10.5)	1.63 [1.10, 2.41]	0.01	1.43 [0.97, 2.11]	0.07
**Major postpartum haemorrhage**	4 (2.9)	42 (2.6)	1.09 [0.40, 2.98]	0.87	0.96 [0.35, 2.66]	0.94
**Postpartum pyrexia**	3 (2.1)	13 (0.8)	2.63 [0.76, 9.12]	0.13	2.33 [0.66, 8.17]	0.19
**Maternal length of stay**^a ^(days)	3.5 ± 2.0	3.1 ± 1.7	0.4 [0.1, 0.7]	0.01	0.3 [-0.0. 0.6]	0.06

^a ^Value is mean ± standard deviation, and the comparison is mean difference (95% CI).

The rate of induction of labour was similar and the overall caesarean section rate did not differ between groups. In the unadjusted analyses significantly more women with BGDM gave birth by caesarean section for fetal distress (RR 1.63, [95%CI 1.10, 2.41], p = 0.01) compared with women with a normal OGCT although this was not significant in the adjusted analyses. The length of postnatal hospital stay was significantly longer (mean difference 0.4 days, [95%CI 0.1, 0.7], p = 0.01) for women with BGDM compared to women with normal OGCT in the unadjusted analyses but not when adjusted for maternal age and BMI (Table 2).

Overall there was no difference in the risk of death or infant adverse outcome between the two groups (Table 3). In unadjusted analyses infants born to women with BGDM were at increased risk of being born preterm (RR 1.68, [95%CI 1.00, 2.80], p = 0.05) and were also significantly more likely to be macrosomic (birthweight ≥4.5 kg) (RR 2.53, [95%CI 1.06, 6.03], p = 0.04) compared with infants born to women with a normal OGCT. When adjusted for maternal age and BMI the association with an earlier gestational age at birth and the risk of being macrosomic remained for infants born to women with BGDM compared with infants born to women with a normal OGCT (Table 3).

**Table 3:** Clinical outcomes among babies born to women with borderline GDM compared with women with a normal OGCT

**Outcome**	**Borderline GDM**	**Normal OGCT**	**Unadjusted relative risk ** [95% CI]	**p value**	**Adjusted relative risk ** [95% CI]	**p value**
***Births***	n = 140 (%)	n = 1596 (%)				
**Infant death or adverse outcome**	18 (12.9)	162 (10.2)	1.27 [0.80, 2.00]	0.31	1.25 [0.79. 1.98]	0.34
**Stillbirth**	1 (0.7)	13 (0.8)	0.88 [0.12, 6.65]	0.90	0.79 [0.10, 6.08]	0.82
**Neonatal death**	0	5 (0.3)	--	--	--	--
**Perinatal death**	1 (0.7)	18 (1.1)	0.63 [0.09, 4.71]	0.66	0.56 [0.07, 4.21]	0.57
**GA at birth**^a ^(weeks)	39.7 (38.5–40.9)	40.1 (39.0–41.0)	--	0.004	--	0.003
**Preterm birth **(GA <37 weeks)	15 (10.7)	102 (6.4)	1.68 [1.00, 2.80]	0.05	1.64 [0.97, 2.75]	0.06
**Very preterm birth **(GA <34 weeks)	4 (2.9)	33 (2.1)	1.38 [0.50, 3.84]	0.54	1.40 [0.50, 3.91]	0.53
**Extremely preterm birth **(GA <28 weeks)	0	12 (0.8)	--	--	--	--
**Apgar 5 minute <7**	3 (2.1)	33 (2.1)	1.04 [0.32, 3.33]	0.95	1.05 [0.32, 3.40]	0.94
**Birthweight**^b ^(g)	3375 ± 626.3	3388 ± 593.4	-13.0 [-116, 89.5]	0.80	-28.8 [-132, 73.9]	0.58
**Birth length**^b ^(cm)	50.2 ± 2.5	50.3 ± 3.3	-0.06 [-0.6, 0.5]	0.82	-0.09 [-0.7, 0.5]	0.75
**Birth head circumference**^b ^(cm)	34.3 ± 1.8	34.4 ± 1.9	-0.10 [-0.4, 0.2]	0.55	-0.17 [-0.5, 0.2]	0.34

***Liveborns***	n = 139 (%)	n = 1583 (%)				
**SFGA **(Birthweight <10^th ^percentile)	10 (7.2)	153 (9.7)	0.74 [0.40, 1.38]	0.35	0.76 [0.41. 1.42]	0.39
**LFGA **(Birthweight ≥90^th ^percentile)	19 (13.7)	153 (9.7)	1.41 [0.91, 2.20]	0.13	1.29 [0.83, 2.00]	0.27
**Macrosomia **(Birthweight ≥4.5 kg)	6 (4.3)	27 (1.7)	2.53 [1.06, 6.03]	0.04	2.27 [0.97, 5.34]	0.06
**Length of stay**^a ^(days)	3 (3–5)	3 (2–4)	--	0.001	--	0.01
**Admission to nursery**	56 (40.3)	450 (28.4)	1.42 [1.14, 1.76]	0.002	1.35 [1.09, 1.68]	0.01
**Admission to NICU**	9 (6.5)	47 (3.0)	2.18 [1.09, 4.36]	0.03	2.05 [1.02, 4.13]	0.04
**RDS**	0	14 (0.9)	--	--	--	--
**Mechanical ventilation**	2 (1.4)	33 (2.1)	0.69 [0.17, 2.85]	0.61	0.65 [0.16, 2.71]	0.56
**Antibiotics <48 hours**	14 (10.1)	78 (4.9)	2.04 [1.19, 3.51]	0.01	2.14 [1.24, 3.68]	0.01
**Sarnat stage 2 or 3 encephalopathy**	18 (12.9)	124 (7.8)	1.65 [1.04, 2.63]	0.03	1.69 [1.06, 2.69]	0.03

^a ^Values are median (IR range). ^b ^Value is mean ± standard deviation, and the comparison is mean difference (95% CI). GA, gestational age; SFGA, small for gestational age; LFGA, large for gestational age; NICU, neonatal intensive care unit; RDS, respiratory distress syndrome

The hospital stay was significantly longer (unadjusted p = 0.001, adjusted p = 0.01) for infants born to BGDM mothers compared with infants born to mothers with a normal OGCT (Table 3). Infants born to BGDM mothers were more than twice as likely to be admitted to NICU (unadjusted p = 0.03, adjusted p = 0.04) and more likely to be admitted to the neonatal nursery (unadjusted p = 0.002, adjusted p = 0.01). Antibiotic use less than 48 hours after birth was significantly greater among infants born to BGDM mothers (unadjusted and adjusted p = 0.01) and more infants born to the BGDM women had Sarnat stage 2 or 3 encephalopathy (unadjusted and adjusted p = 0.03) compared with infants born to women with a normal OGCT (Table 3).

## Discussion

In this cohort of primiparous women in Australia, 8% were found to have BGDM. In this study, associations with BGDM were identified for maternal obesity and increasing maternal age, similar to those identified for gestational diabetes in other literature [16-18].

In our study, women with BGDM had a higher risk of adverse health outcomes overall, and were more likely to develop pregnancy induced hypertension, require a caesarean section for fetal distress and have a longer postnatal hospital stay. However, we did not detect a statistically significant increase in the risk of pre-eclampsia or caesarean section overall among women with BGDM, which has been reported by previous studies [9-11]. Increasing maternal age and BMI are strongly associated with adverse maternal health outcomes. When these factors were adjusted for, no differences were seen for health outcomes between women with BGDM and normal women.

We identified an increased risk of preterm birth amongst BGDM mothers. The reason for this is not readily apparent, given that there is no difference in the rate of induction of labour between the two groups. Infants of BGDM mothers were more likely to require a NICU and/or nursery admission and longer hospital stays. This may be explained by the higher rate of pregnancy induced hypertension, caesarean section for fetal distress, preterm birth and encephalopathy (Sarnat stage 2 or 3) in this group. Infants born to BGDM mothers in both unadjusted analyses and when adjusted for maternal age and maternal BMI were also at higher risk of macrosomia, which is consistent with previous studies [10,11].

Our study has identified increased risks of maternal adverse health outcomes overall and a range of infant adverse health outcomes associated with BGDM. In Australia, there are over 250,000 births annually [19]. Our data suggest that a substantial number of Australian pregnant women, over 20,000 each year, will have BGDM and therefore maternal and infant adverse health outcomes that are directly or indirectly attributable to BGDM. Evidence from the Australian Carbohydrate Study in Pregnant Women (ACHOIS) trial [20] confirmed that untreated mild GDM is associated with relatively rare but nonetheless significant adverse perinatal outcomes. The trial demonstrated that the risk of these outcomes can be reduced with standard treatment consisting of individual dietary and lifestyle advice during pregnancy. There is, however, insufficient evidence regarding the benefits and harms of similar intervention for women with BGDM, with only one small clinical trial identifying a significantly reduced risk of large-for-gestational age infants with dietary advice and regular blood glucose monitoring for women with borderline glucose intolerance [21]. Data from our analysis highlight the need for well-designed large randomised clinical trials to investigate the benefits and harms of such treatment for women with BGDM.

## Competing interests

The authors declare that they have no competing interests.

## Authors' contributions

All authors contributed to the study design, interpretation of the data and preparation of the drafts of the manuscript. In addition CAC and ARR coordinated the study and the collection of data. KJW performed the data analyses. All authors read and approved the final manuscript.

## Pre-publication history

The pre-publication history for this paper can be accessed here:


